# It Is Time to Study Overlapping Molecular and Circuit Pathophysiologies in Alzheimer’s and Lewy Body Disease Spectra

**DOI:** 10.3389/fnsys.2021.777706

**Published:** 2021-11-18

**Authors:** Noritaka Wakasugi, Takashi Hanakawa

**Affiliations:** ^1^Integrative Brain Imaging Center, National Center of Neurology and Psychiatry, Tokyo, Japan; ^2^Department of Integrated Neuroanatomy and Neuroimaging, Graduate School of Medicine, Kyoto University, Kyoto, Japan

**Keywords:** Alzheimer’s disease, Parkinson’s disease, MRI, functional connectivity, fluid biomarkers, PET, SPECT, longitudinal cohort design

## Abstract

Alzheimer’s disease (AD) is the leading cause of dementia due to neurodegeneration and is characterized by extracellular senile plaques composed of amyloid β_1__–__42_ (Aβ) as well as intracellular neurofibrillary tangles consisting of phosphorylated tau (p-tau). Dementia with Lewy bodies constitutes a continuous spectrum with Parkinson’s disease, collectively termed Lewy body disease (LBD). LBD is characterized by intracellular Lewy bodies containing α-synuclein (α-syn). The core clinical features of AD and LBD spectra are distinct, but the two spectra share common cognitive and behavioral symptoms. The accumulation of pathological proteins, which acquire pathogenicity through conformational changes, has long been investigated on a protein-by-protein basis. However, recent evidence suggests that interactions among these molecules may be critical to pathogenesis. For example, Aβ/tau promotes α-syn pathology, and α-syn modulates p-tau pathology. Furthermore, clinical evidence suggests that these interactions may explain the overlapping pathology between AD and LBD in molecular imaging and post-mortem studies. Additionally, a recent hypothesis points to a common mechanism of prion-like progression of these pathological proteins, via neural circuits, in both AD and LBD. This suggests a need for understanding connectomics and their alterations in AD and LBD from both pathological and functional perspectives. In AD, reduced connectivity in the default mode network is considered a hallmark of the disease. In LBD, previous studies have emphasized abnormalities in the basal ganglia and sensorimotor networks; however, these account for movement disorders only. Knowledge about network abnormalities common to AD and LBD is scarce because few previous neuroimaging studies investigated AD and LBD as a comprehensive cohort. In this paper, we review research on the distribution and interactions of pathological proteins in the brain in AD and LBD, after briefly summarizing their clinical and neuropsychological manifestations. We also describe the brain functional and connectivity changes following abnormal protein accumulation in AD and LBD. Finally, we argue for the necessity of neuroimaging studies that examine AD and LBD cases as a continuous spectrum especially from the proteinopathy and neurocircuitopathy viewpoints. The findings from such a unified AD and Parkinson’s disease (PD) cohort study should provide a new comprehensive perspective and key data for guiding disease modification therapies targeting the pathological proteins in AD and LBD.

## Introduction

In parallel with global aging, the number of elderly patients with neurodegenerative diseases is rapidly increasing worldwide. Among others, Alzheimer’s disease (AD) is the most common, followed by Parkinson’s disease (PD) (Hou et al., 2019). AD is the most common cause of dementia (Jansen et al., 2015). People with AD have memory impairment and deficits of self-awareness as the core clinical symptoms. People with AD also exhibit behavioral and psychological symptoms of dementia (BPSD), including anxiety, depression and hallucinations (Jack et al., 2018). AD has key neuropathological hallmarks, including extracellular senile plaques composed mainly of amyloid β_1__–__42_ (Aβ_1__–__42_) and intracellular neurofibrillary tangles (NFTs) consisting of phosphorylated tau (p-tau) (Buckner et al., 2005; Jansen et al., 2015; Teipel et al., 2015; Demirtaş et al., 2017; Ishii et al., 2019). Abnormal Aβ and tau are considered key molecules in the etiopathogenesis of AD. However, it remains unclear how disease-specific the abnormal Aβ and tau proteins are in AD because these molecules are also involved in the pathophysiology of geriatric diseases other than AD (Galvin et al., 2011). Now it is widely recognized that AD is a disease spectrum spanning from preclinical AD, amnestic mild cognitive impairment (aMCI) and fully developed AD (Weiner et al., 2017).

Parkinson’s disease is the second most common neurodegenerative disorder, characterized by motor symptoms, including akinesia, rigidity, and resting tremor. Non-motor symptoms include cognitive impairment, depression, anhedonia, anxiety, visual hallucination, and pain (Postuma et al., 2015). Moreover, People with PD usually have autonomic dysfunctions, including constipation, urinary disturbance, and orthostatic hypotension (Postuma et al., 2015). In PD, Lewy neurites, neurites with accumulations of protein aggregates mainly composed of α-synuclein (α-syn), are present in both the central and peripheral nervous systems. These protein accumulations are considered to give rise to the various neurological symptoms (Henderson et al., 2019; Mendoza-Velásquez et al., 2019). Furthermore, it is known that systemic symptoms of PD not only coexist with motor symptoms, but also appear before the onset of motor symptoms (Heinzel et al., 2019). Currently, dementia in advanced PD is commonly referred to as PD dementia (PDD). As part of the PD spectrum, dementia with Lewy bodies (DLB) may start from cognitive decline leading to dementia before or almost at the same time as the development of parkinsonism. Thus, PD is also a disease spectrum spanning from prodromal PD without motor symptoms to fully developed PD/PDD and DLB (Heinzel et al., 2019), which may be comprehensively called Lewy body disease (LBD), a synonym of the PD spectrum here, from an etiopathological perspective.

Alzheimer’s disease and PD spectra include many common cognitive and psychiatric symptoms and risk factors. Recently, the core pathology of AD and LBD (i.e., PD/DLB) has been hypothesized to share the same mechanism, called the protein propagation hypothesis (Dagher and Zeighami, 2018; Colin et al., 2020). The hypothesis posits that neurodegeneration progresses by cross-synaptic transmission of abnormal proteins with altered conformation. This could explain the observation that in both PD and AD, various clinical signs gradually appear with aging, which allows the propagation of abnormal proteins. Even if this hypothesis is correct, many issues remain unresolved, such as differences in the neural circuits involved. Furthermore, the coexistence of AD and PD pathology is common in sporadic cases (Irwin et al., 2013). This coexistence may be the key to elucidating the molecular pathology of sporadic cases of LBD and AD.

In previous studies, AD and PD cases have typically been enrolled in different and independent cohorts. The Alzheimer’s Disease Neuroimaging Initiative (ADNIs) is a series of cohort studies of AD (Petersen et al., 2010; Weiner et al., 2017; Veitch et al., 2019), as The Parkinson Progression Markers Initiative is for PD (Marek et al., 2011). However, considering the shared pathological mechanisms, many important questions may only be resolved by studies that include both AD and PD spectra in the same cohort. For example, mild cognitive impairment (MCI) may herald dementia (Flicker et al., 1991). MCI initially drew attention in the field of AD research. However, the background pathology of MCI is not limited to AD, but includes various pathological conditions such as PD (Dichgans and Leys, 2017; Baiano et al., 2020). A comprehensive cohort including different types of MCIs will likely cast new light on the prodromal stage of dementia.

In this article, we review basic research on the distribution and interactions of pathological proteins in the brains of AD and LBD, after briefly summarizing their clinical and neuropsychological manifestations. We also describe functional and connectivity changes in the brain in AD and LBD. Finally, we will argue for the necessity for a neuroimaging cohort treating the AD spectrum and the PD spectrum as a continuum. The findings, which will be obtained from the proposed cohort study, should provide a new comprehensive perspective for these two most important neurodegenerative disorders.

## Alzheimer’s Disease

Alzheimer’s disease is the most common dementia in the population over 65 years of age. The core clinical symptom of AD is impaired recent memory, which supposedly results from dysfunctions of the medial temporal lobe (MTL) and the hippocampus. Most patients with AD first present with impaired episodic memory, followed by more extensive cognitive dysfunctions, including amnesia, apraxia, and visuospatial deficits (Eramudugolla et al., 2017). These cognitive symptoms are ascribed to the dysfunctions of the temporal lobe, parietal lobe, and occipital lobe. Semantic memory problems caused by lateral temporal lobe dysfunction and insight and spontaneity deficits resulting from frontal lobe dysfunction may also be observed (Hodges and Patterson, 1997; Boublay et al., 2020).

### Diagnostic Criteria

Alzheimer’s disease is diagnosed primarily by clinical history. Multiple markers, including cerebrospinal fluid (CSF), neuroimaging, and genetic tests, are used as supportive findings (Jack et al., 2018). For the clinical diagnosis of AD, the following two criteria are used: (i) finding of major neurocognitive disorder (NCD), as defined by the fifth edition of the Diagnostic and Statistical Manual of Mental Disorders (DSM-5) (Eramudugolla et al., 2017), and (ii) the diagnostic guidelines of the National Institute on Aging and Alzheimer’s Association (NIA-AA) (Jack et al., 2018). The common item in the two criteria is slowly progressive dementia.

DSM-5 criteria make use of genetic risks, including family history and identification of the causative genes. Cognitive dysfunctions need to be shown in two or more cognitive domains centered on the memory/learning disorders. NIA-AA criteria also consider the genetic risks and clinical symptoms, similar to DSM-5. NIA-AA also takes into consideration evidence for the existence of AD pathology indicated by fluid biomarkers such as CSF and imaging biomarkers such as positron emission tomography (PET).

For research purposes, the International Working Group-2 (IWG-2) criteria for AD may be used (Dubois et al., 2014). The criteria broadly classify AD into typical AD, in which patients initially develop episodic memory impairment, and atypical AD, in which patients develop unique symptoms such as logopenic aphasia and frontal lobe signs. In addition, the evaluation of Aβ and tau, using CSF or PET, is also essential.

Mild cognitive impairment is a condition where slight cognitive impairment is recognized objectively compared with a condition before illness, but the patients’ activities of daily living are independent. MCI centered on memory deficits is clinically diagnosed as amnestic MCI (aMCI) presumably caused by AD pathology (Petersen et al., 1999, 2001). In addition, if there are findings from fluid/imaging markers indicating abnormalities in Aβ/tau, this condition is defined as AD-MCI (Albert et al., 2011).

### Neuropsychological Testing

Neuropsychological tests for AD are used for screening, differential diagnosis, and severity assessment. In clinical practice, screening for dementia or MCI is performed by tests for general cognitive functions. Mini Mental State Examination (MMSE), a simple test for assessing multiple cognitive domains, is used with a cutoff value of 23/24 for dementia (Trivedi, 2017). To diagnose MCI, Montreal Cognitive Assessment (MoCA) may be used with a cutoff value of 25/26 (Davis et al., 2015). These tests are helpful not only for screening through the assessment of total scores, but also for evaluating cognitive subcategories, including memory, attention, and visuospatial cognition.

As the disease progresses, patients with AD who initially had only memory impairment begin to show impairment involving multiple cognitive domains, including visuospatial cognition and language. Therefore, the Wechsler Adult Intelligence Scale (WAIS) and the Wechsler Memory Scale (WMS-R) are performed for a more detailed and comprehensive evaluation. However, because these advanced tests take more than 1 h, they are not suitable for screening.

Differential diagnosis to estimate the background pathology of dementia cannot solely rely on the neuropsychological examination, and is accordingly usually performed in combination with the assessment of clinical course and diagnostic imaging. In clinical practice, it is important to distinguish AD from other dementias such as DLB. Typical early symptoms are predominantly memory deficits in AD; executive function and visuospatial cognition are impaired only after memory deficits become apparent (Jack et al., 2018). Therefore, for differential diagnosis of AD, patients should undergo tests of memory, attention, executive functions, and visuospatial cognition to distinguish from DLB, in which executive function and visuospatial cognition are impaired at the early stage. However, because half of the cases of DLB are complicated by AD pathology, the possibility that DLB patients have AD-like memory impairment from the onset needs to be taken into account (Yoshizawa et al., 2013).

The severity assessment is essential for selecting rehabilitation and welfare services, and for longitudinal cognitive assessment in clinical research. Clinical Dementia Rating (CDR) (Morris, 1993) is a scale that evaluates general cognitive functions and community affairs, home/hobbies, and personal care. CDR yields a global scale (CDR-GS) and a sum of boxes (CDR-SB). CDR-GS is a general severity calculated by weighting the results of each subscale and is commonly used for disease staging. On the CDR, CDR-GS 0.5 corresponds to MCI, and 1.0 or larger corresponds to dementia (Chang et al., 2011). CDR-SB provides more information in terms of quantitative evaluation than CDR-GS, especially in MCI and early AD (O’Bryant et al., 2010). A feature of CDR is that it also refers to interviews with family members. Thus, CDR provides accurate information for understanding disease severity and significantly impaired function when evaluated by a proficient examiner that is well informed by the caregiver. Alzheimer’s Disease Assessment Scale-Cognitive Subscale (ADAS-cog) is an assessment of 11 cognitive subcategories related to AD symptoms (Kueper et al., 2018). It is time-consuming, yet suitable for observing changes in cognitive function over time and is often used in clinical trials.

### Genetics

Most AD cases are sporadic, and the most prominent risk factor is aging (Angelova and Brown, 2019). AD also has familial cases, and many genetic risk factors are known. In autosomal dominant AD, mutations in proteins related to Aβ production, such as presenilin 1/2 (*PSEN1/PSEN2*) and amyloid precursor protein (*APP*) have been reported (Robinson et al., 2017). The mutation of *PSEN1* accounts for the most common form of familial AD. *PSEN1* is a component of the γ-secretase complex, which plays an essential role in the production of Aβ. *APP* mutations were first reported as an etiological factor in intracranial hemorrhage with amyloid pathology (amyloid angiopathy) and subsequently identified in familial AD cases. Patients with autosomal dominant AD (AD-AD) with *PSEN1/PSEN2* and *APP* mutations may exhibit predominantly motor symptoms of akinetic-right type caused by Aβ deposition in the basal ganglia (Vöglein et al., 2019). Therefore, it may be difficult to distinguish some AD-AD cases from patients with PDD and DLB.

The ε*4* allele of the *APOE* gene is the most important risk factor for sporadic AD (Jansen et al., 2015), being present in more than half of all AD cases, compared with a frequency of ∼14% in the general population (Pontifex et al., 2018). The ApoE protein functions as a ligand for the low-density lipoprotein (LDL) receptor family on the cell, and its abnormality causes atherosclerosis. In the brain, ApoE is mainly produced by astrocytes and is involved in transporting and removing cholesterol (Hauser et al., 2011). Furthermore, ApoE was reported to be involved in the degradation of Aβ (Jiang et al., 2008). Humans carry the ε*2*,ε*3*, and ε*4* alleles with a worldwide frequency of 8.4, 77.9 and 13.7%, respectively (Liu et al., 2013). Compared with ε*3/*ε*3*, ε*4* hetero carriers have an odds ratio (OR) of 2.8 [95% confidence interval (CI) 2.3–3.5], whereas homo carriers (ε*4/*ε*4*) have an OR of 11.8 (95% CI 7.0–19.8) (Pontifex et al., 2018). Conversely, *APOE*ε*2* is considered a protective factor for AD symptoms and pathology (Liu et al., 2013; Jindal and Bansal, 2016). *APOE*ε*4* has been reported to be involved in abnormal Aβ accumulation and perturbed functional connectivity (FC) (Sheline et al., 2010a).

Recent whole-exome sequencing and genome-wide association studies (GWAS) revealed a rare variant of the triggering receptor expressed on myeloid cells 2 (*TREM2*) in AD (Guerreiro et al., 2013; Jonsson et al., 2013). Interestingly, TREM2, which is expressed by microglia, could be involved in the removal of Aβ. It is expected that advances in genetic research will continue to reveal novel AD risk genes.

### Fluid Biomarkers

Aβ and tau, which constitute the hallmark senile plaques and NFTs, respectively, are the most important biomarkers of AD (Hampel et al., 2010; Olsson et al., 2016). The Alzheimer’s Disease Neuroimaging Initiative (ADNI) revealed that CSF can be used to detect the earliest changes in AD pathology (Weiner et al., 2012). Another study found decreased CSF-Aβ more than 25 years before the onset of AD (Bateman et al., 2012). These findings are incorporated into clinical diagnostic criteria, such as the NIA/AA criteria, for preclinical AD, which is the prodromal stage with normal cognition before MCI. As such, CSF-Aβ is considered a promising AD biomarker for early diagnosis, and is widely used in both the clinical and research laboratory settings. Lower Aβ_1__–__42_ level and higher total tau (t-tau) or p-tau are reliable CSF biomarkers of AD pathology. Also, several reports suggest that the ratio of Aβ_1__–__42_/Aβ_1__–__40_ (Blennow and Hampel, 2003), t-tau/Aβ_1__–__42_ (Shaw et al., 2009) or p-tau/Aβ_1__–__42_ (De Meyer et al., 2010) more accurately reflect AD pathology than Aβ_1__–__42_ or tau alone. In p-tau, CSF-tau phosphorylated at serine 181 (p-tau 181) is regarded as an AD-specific biomarker (Hampel and Teipel, 2004). As a CSF marker under development, Aβ oligomers might become a useful biomarker. Aβ oligomers are formed during the polymerization process of Aβ fibrils and are suggested to cause synaptic dysfunction. However, a hurdle to quantitative evaluation is the limited amount of Aβ oligomers in CSF (Hampel et al., 2010).

It has been suggested that plasma p-tau 181 is a potential AD-specific marker for predicting neurodegeneration and cognitive decline (Barthélemy et al., 2020; Moscoso et al., 2021). The results of a meta-analysis of blood biomarkers showed increased t-tau, p-tau 181, p-tau 217, neurofilament light protein (NFL) and decreased Aβ_1__–__42_ in patients with AD and AD-MCI (Qu et al., 2021).

### Neuroimaging

#### Blood Flow/Metabolism Changes

Detecting changes in cerebral perfusion using single-photon emission computed tomography (SPECT) is widely used in the clinical field to distinguish AD from other dementias. ^18^F-fluorodeoxyglucose positron emission tomography (FDG PET) detects patterns of regional glucose metabolism associated with AD pathology (neuronal loss). The core finding is similar between perfusion SPECT and FDG-PET. Decreased perfusion or glucose metabolism in the temporal lobe, the parietal lobe and the posterior cingulate gyrus (PCC) is indicative of AD (Bloudek et al., 2011). The sensitivity of perfusion SPECT to distinguish patients with AD from healthy subjects is 80%, and the specificity is 85% (McKhann et al., 2011). FDG-PET discriminates between AD and healthy subjects with a sensitivity of 90% and a specificity of 89% (Bloudek et al., 2011).

#### Amyloid Positron Emission Tomography

Visualization of deposition of Aβ or tau in the brain is strong evidence of AD pathology. Both amyloid-PET and tau-PET provide indispensable information for the classification and interpretation of the underlying pathophysiology as well as for the early diagnosis. Recently, the removal of Aβ fibrils and tau accumulation is considered a promising disease-modifying therapy, and thus the visualization of abnormal Aβ or tau in the brain is becoming even more important from the viewpoint of treatment. Aβ deposition can be visualized using PET. There are several standard probes, including Pittsburgh compound B (^11^C-PiB) and several ^18^F agents (florbetapir and florbetaben), for amyloid-PET (Chételat et al., 2020). ^11^C-PiB has been used as a standard PET probe for a long time and has substantially advanced our knowledge of the disease. However, because ^11^C-PiB has a half-life of only 20 min, its use is limited to hospitals with a cyclotron and synthesis unit. Currently, multiple ^18^F-probes, which have a longer half-life of 110 min, and is thus deliverable from a factory, have been developed so that amyloid-PET can be performed at more hospitals than before.

Both CSF-Aβ and amyloid-PET are tests for identifying AD pathology related to senile (amyloid) plaques. In a report comparing PiB-PET and CSF biomarkers, PiB-positive patients had significantly lower Aβ and significantly higher t-tau and p-tau in CSF compared with PiB-negative subjects (Shimada et al., 2011). There is a negative correlation between PET Aβ levels and CSF Aβ levels. The diagnostic value of amyloid-PET and CSF-Aβ is comparable, but they differ slightly in the information they provide. Unlike CSF-Aβ, amyloid-PET can visualize the spatial extent of amyloid deposition. In AD, the core imaging findings are amyloid accumulation in the frontal lobe, PCC and precuneus. The amyloid burden in these regions can explain the pathophysiology of AD well and can provide a key to differential diagnosis from other dementias. PET probe injection is less invasive than a lumbar puncture, so that cognitively healthy people may undergo amyloid-PET more comfortably than CSF collection for screening or possibly prodromal interventions. Conversely, CSF-Aβ is suggested to reflect AD pathology earlier than amyloid-PET (Mattsson et al., 2015). Even if amyloid-PET is negative, CSF measurements may identify earlier amyloid pathology. Furthermore, if a promising CSF biomarker is developed in the future (Hampel et al., 2010), additional tests can be performed if the frozen CSF sample is preserved. Thus, past patient conditions can be evaluated using the latest testing method with CSF. CSF and amyloid-PET are thus complementary, and it is important to combine the two tests to assess amyloid pathology more comprehensively than before.

#### Tau Positron Emission Tomography

Although tau-PET has faced some problems, such as difficulty in developing a probe with a high affinity for AD-tau and a low affinity for Aβ_1__–__42_ (Valotassiou et al., 2018), multiple nuclides, including ^11^C-PBB3/^18^F-PM-PBB (Maruyama et al., 2013; Tagai et al., 2021) and ^18^F-flortaucipir (Leuzy et al., 2019) are now available. The intracerebral accumulation rate of tau-PET nuclides correlates well with the severity of the disease. Therefore, it attracts attention as a modality with favorable characteristics compared with other biomarker candidates (Joie et al., 2020).

Tau-PET is a promising imaging method for the diagnosis of various neurodegenerative disorders including the AD spectrum. Amyloid burden visualized by PET is affected by aging and the *ApoE* allele, and hence the specificity of AD diagnosis with amyloid PET is relatively low especially in the elderly aged over 80 years old (Ossenkoppele et al., 2015). Contrastingly, tau-PET has been reported to show high specificity in differentiating AD from other neurodegenerative disorders (Ossenkoppele et al., 2018). Moreover, tau-PET positivity can be a better predictor of cognitive decline in AD as compared with CSF-p-tau 181 or amyloid PET (Bucci et al., 2021). Another study using ^11^C-PBB3 PET revealed cases with tau accumulation without Aβ_1__–__42_ accumulation by amyloid-PET (Shimada et al., 2017). This amyloid-negative and tau-positive state may represent an entity called primary age-related tauopathy (PART) (Crary et al., 2014). Dementia with PART overlaps with senile dementia of the NFT type (SD-NFT), which is tau-related dementia distinct from AD. Patients with SD-NFT are sometimes misdiagnosed as AD, and hence tau-PET may be useful for differential diagnosis.

The PET can provide data most closely related to the neuropathological diagnosis. Yet, PET is an expensive tool and thus may not be affordable in many places in the world. Therefore, early biomarkers using relatively easy-to-perform tests, such as blood tests and magnetic resonance imaging (MRI), should be explored. Until such surrogate markers become available, PET will remain one of the most important tools in cohort studies of neurodegenerative disorders. In the meantime, it is important to organize comprehensive and high-quality cohort studies to acquire multiple PET scan along with fluid samples (both blood and CSF) and MRI.

#### Structural Magnetic Resonance Imaging

Hippocampal and MTL atrophy in structural MRI is frequently used as an adjunct to clinical diagnosis of AD. In a systematic review, MTL atrophy was discriminative between AD and healthy persons with a sensitivity of 85% and a specificity of 88% (Scheltens et al., 2002). In AD, the accumulation of Aβ_1__–__42_ and p-tau is thought to become saturated before the onset of symptoms such as cognitive decline and cerebral atrophy, which become apparent later (Weiner et al., 2015). Reflecting this time course, changes in brain structure occur mainly after the onset of cognitive decline, and therefore, structural MRI primarily provides imaging markers for diagnosis and severity assessment after onset.

Reduced hippocampal volume is a consistent finding across structural MRI studies as previously observed in postmortem brains. A voxel-based morphometry (VBM) analysis of structural MRI suggested that the progression of cerebral atrophy in AD parallels the putative propagation of neurofibrillary tangle implicated by Braak staging (Matsuda, 2016). A combining a neuropsychological battery with VBM analysis of MRI data from ADNI found correlations between memory loss and mediolateral temporal lobe atrophy as well as between executive dysfunction and parietotemporal atrophy (Nho et al., 2012). White matter changes in AD are reported to be less significant than gray matter changes. However, a meta-analysis of VBM suggests that in patients with AD, there is considerable volume loss in the white matter beneath the parahippocampal gyrus and in the posterior corpus callosum (Matsuda, 2016).

#### Diffusion Tensor Imaging

Tau is a microtubule-associated protein that is involved in stabilizing the microtubule structure of neuronal axons (Guo et al., 2017). DTI is a method for capturing changes in the microstructure of the white matter, and it may be able to detect axonal changes related to tau pathology. In AD, reduced fractional anisotropy (FA) is most pronounced in fiber tracts in the limbic area, such as the fornix, and in the temporal lobe (Teipel et al., 2015). This abnormality in white matter microstructure is an early change seen in the process of AD neurodegeneration. Moreover, an FA reduction in PCC has been reported in patients in the prodromal stage, including subjective cognitive impairment (SCI) and mild MCI (Stenset et al., 2011).

#### Functional Magnetic Resonance Imaging

Functional MRI can be used to observe brain activities non-invasively via blood-oxygen-level-dependent (BOLD) signals, which reflect local fluctuations in the oxygenation of cerebral blood (Ogawa et al., 1990). Task-related changes in BOLD signals and synchronous BOLD fluctuations across multiple regions at resting state exhibit changes associated with the onset and progression of neurodegenerative diseases, including AD. In fMRI studies with a memory task, patients with early MCI show increased activation in the MTL (Dickerson et al., 2004, 2005; Chhatwal and Sperling, 2012) and hippocampus (Celone et al., 2006), compared with healthy controls. This hyperactivation may be lost at follow-up (O’Brien et al., 2010; Bai et al., 2011). Therefore, it is hypothesized that temporary hyperactivation is a compensatory mechanism in the early stage of AD (Clment and Belleville, 2010).

**FIGURE 1:**
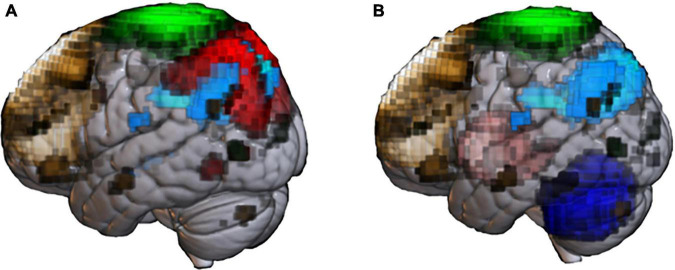
Abnormal resting-state networks in Alzheimer’s disease (AD) and Parkinson’s disease (PD). Functional network templates were extracted from the MRI database (70 healthy elderly) at the National Center of Neurology and Psychiatry’ to display network abnormality reported in past AD **(A)** and PD **(B)** studies. **(A)** Default mode network (DMN, light blue) is the representative network impaired in patients with senile-onset AD. Early- onset AD shows impaired not only DMN, but also executive control network (brown), sensory-motor network (SMN, green) and dorsal-attention network (red). **(B)** Patients with PD shows extensive network impairment, including basal ganglia (pink), cerebellum network (blue), and SMN. PD patients with cognitive impairment shows impaired DMN and ECN.

Resting-state functional connectivity MRI (rsfcMRI) is a promising imaging modality for pre-symptomatic diagnosis of AD. This type of MRI focuses on the co-fluctuation of BOLD signals across brain regions in the low-frequency band below 0.1 Hz (Liu, 2013). Thus, rsfcMRI studies assume that brain regions with mutually interwoven networks should show similar fluctuations in low-frequency BOLD signals, reflecting concerted fluctuation of synaptic activity propagated through the neural network. Another assumption is that the level of coordination of signals across brain regions reflect specific functions subserved by the network. Interestingly, FC can be detected between regions without known direct fiber connections (Honey et al., 2009), meaning that rsfcMRI may detect network abnormalities that cannot be discovered by studies of structural connectivity. Thus, rsfcMRI is valuable as a biomarker of AD because brain networks are differentially affected by various factors, including the stage of the disease (Wang et al., 2006; Sheline and Raichle, 2013; Kim et al., 2015; Córdova-Palomera et al., 2017; Palmqvist et al., 2017), symptoms (Adriaanse et al., 2014), treatments (Ochmann et al., 2017), pathology (Sheline et al., 2010a,b; Robinson et al., 2017; Shi and Holtzman, 2018) and genetic factors (Vöglein et al., 2019). In AD, various types of network alterations, including those of the default mode network (DMN), are reported (Figure 1A).

The most well-established finding is that hippocampal–default mode network (DMN) connectivity is reduced compared with healthy people (Hohenfeld et al., 2018). Among the nodes within the DMN, the importance of connectivity between the precuneus and PCC has been demonstrated in numerous studies (Greicius et al., 2003; Wu et al., 2011; Teipel et al., 2015; Palmqvist et al., 2017; Ibrahim et al., 2021). The precuneus plays a central role in visuospatial imagery, episodic memory retrieval, and processing of self-related information (Cavanna and Trimble, 2006). The PCC is related to internally-directed thinking and helps allocate attention efficiently in collaboration with the ECN (Leech et al., 2011). Abnormal connectivity between the PCC and precuneus likely reflects pathological changes in AD as substantial abnormalities are detected in these regions, such as decreased glucose metabolism and perfusion, and accumulation of abnormal proteins (Bloudek et al., 2011; Daerr et al., 2017). Indeed, the FC abnormality in AD is, at least in part, associated with amyloid accumulation. Changes in AD-like connectivity have been found in amyloid-positive non-AD patients (Sheline et al., 2010b). Surprisingly, *APOE*ε*4* carriers have reduced connectivity at the stage of no amyloid accumulation (Sheline et al., 2010a).

The pattern of DMN FC abnormality may depend on the age of onset. Early-onset AD shows impairment among a wide range of connectivities, including visual network, auditory network, sensorimotor network (SMN), DMN, executive control network (ECN), and dorsal attention network (DAN), compared with senile-onset AD (Adriaanse et al., 2014).

Several rsfcMRI studies have attempted to classify categories within the AD spectrum (e.g., AD-MCI vs. AD), AD from other dementia spectra, or from healthy elderlies (Zhou et al., 2010; Wee et al., 2016; Zhang et al., 2019). Conventional static FC, dynamic FC (reflecting changes in FC over time), and amplitude of low-frequency fluctuations (ALFF) have been used for classification. The combination of these different modalities slightly improves classification accuracy (de Vos et al., 2018). Machine learning (ML) classification using PCC-FC can classify AD and healthy elderlies with a sensitivity of 75.2% and a specificity of 74.9% (Ibrahim et al., 2021). The ICA-based ML technique can classify AD patients and healthy elderlies with a sensitivity of 78.2% and a specificity of 83.2% (Ibrahim et al., 2021). In classifying AD patients from healthy elderlies, the classification performance is significantly improved when multiple MR modalities (structural MRI, DTI, and rsfMRI) are combined, compared with a single imaging modality (Schouten et al., 2016).

## Parkinson’s Disease

The core condition of the LBD/PD spectrum, PD, is the most common movement disorder and is caused primarily by the loss of nigrostriatal dopaminergic neurons, associated with the intracellular deposition of α-syn (Postuma et al., 2015). The cardinal clinical symptom is a movement disorder comprehensively called parkinsonism, with features of akinesia, rigidity, resting tremor, and postural instability. The PD spectrum may also present with cognitive disturbance and dementia. In PD, the prevalence of MCI is reported to be 40% (Baiano et al., 2020), and that of dementia is 30% (Hanagasi et al., 2017). Dementia in PD spectrum is characterized by hallucination with highly specific content and temporal fluctuations in cognitive function centered on attention and arousal (Dickson et al., 2017). DLB is diagnosed if dementia is already present from the onset, and PD with dementia (PDD) is diagnosed if cognitive decline occurs after the onset of motor symptoms. In general, 1 year after the onset of PD is used as the boundary between PDD and DLB (1-year rule) (Bonanni et al., 2006; McKeith et al., 2017). Thus, PDD and DLB may constitute a spectrum (Jellinger and Korczyn, 2018). Some reports indicate differences in clinical features between PDD and DLB. Patients with DLB tend to have severer visuospatial cognitive, executive, and attentional decline than people with PDD (Morra and Donovick, 2014). In terms of the motor symptoms, resting tremor and laterality are less evident in DLB than in PDD.

Symptoms common in AD, including olfactory dysfunction, apathy, and anxiety depression, are also common in dementia in the PD spectrum (Attems et al., 2014; Le Heron et al., 2018; Galts et al., 2019). As described later, DLB/PDD is associated with various degrees of AD pathology, and this shared pathology may be the reason for the clinical similarity between AD and PDD/DLB.

### Diagnostic Criteria

There are two widely used diagnostic criteria for PD—the United Kingdom Parkinson’s Disease Society Brain Bank (UKPDSBB) clinical diagnostic criteria (Gibb and Lees, 1988; Hughes et al., 1992) and the International Parkinson and Movement Disorder Society (MDS) clinical diagnostic criteria for PD (Postuma et al., 2015). Both require bradykinesia as a core symptom.

UKPDSBB criteria diagnose PD with the following three steps: presence of parkinsonism (step 1), exclusion of other parkinsonian syndromes (step 2), and existence of supportive features (step 3). These criteria are used worldwide and have had their accuracy verified by postmortem pathological diagnoses. The MDS diagnostic criteria include criteria for clinical diagnosis and research criteria for probabilistic prediction of PD before the diagnosis, according to risk factors and mild symptoms. Multiple studies have reported a high concordance rate for PD diagnosis between UKPDSBB criteria and MDS criteria (Malek et al., 2017; Postuma et al., 2018). Neither criterion mentions PDD, which requires an independent assessment (Sachdev et al., 2014).

### Motor and Neuropsychological Tests

In the PD spectrum, the MDS-Sponsored Revision of the Unified Parkinson’s Disease Rating Scale (MDS-UPDRS) is widely used for the evaluation of motor and non-motor symptoms (Goetz et al., 2008). MDS-UPDRS is divided into the following four sections: questionnaire for non-motor symptoms in daily life (Part 1), motor symptoms in daily life (Part 2), motor examination by a physician (Part 3), and questionnaire for motor complications (Part 4). The score is used for evaluation of disease severity and response to treatment.

Because of the difference in clinical symptoms, PD has been traditionally subjected to neuropsychological tests slightly different from those for AD. A condition consistent with MCI is found in nearly half of people with PD (Goldman et al., 2018). A longitudinal study reported that 80% of people with PD eventually show dementia (Williams-Gray et al., 2007). In PD, executive function is particularly vulnerable. Executive dysfunction is found from the prodromal stage and is more pronounced than memory impairment. Therefore, in the cognitive evaluation of PD, a test of frontal lobe functions such as Frontal Assessment Battery (FAB) (Dubois et al., 2000) is often added to a general cognitive test such as MMSE. Otherwise, it is recommended to perform MoCA, in which executive functions are tested in more detail than MMSE (Weintraub et al., 2015).

To assess cognitive dysfunctions in early PD (such as PD-MCI), it is recommended to examine five cognitive domains—memory, executive, attention/working memory, language, and visuospatial function (Litvan et al., 2012). To this end, thorough testing with WAIS and WMS may be considered (Tang et al., 2016).

In PDD, psychiatric symptoms are often directly linked to a decrease in Activities of Daily Living (ADL), and it is essential to evaluate the severity of psychiatric symptoms properly (Rosenthal et al., 2010). MDS-UPDRS Part 1 evaluates these symptoms. Questionnaires such as Beck Depression Inventory (BDI) (Beck et al., 1961), Questionnaire for Impulsive-Compulsive Disorders (QUIP) (Probst et al., 2014), and State-Trait Anxiety Inventory (STAI) (Caillava-Santos et al., 2015) are often used for the assessment of psychiatric symptoms in the PD spectrum.

### Genetics

Like AD, PD is primarily a sporadic disease. Multiple genetic and environmental factors appear to be involved in the onset of PD. Approximately 5% of PD cases are familial, some of which are inherited in a Mendelian fashion, including *SNCA*, *PKRN*, and *LRRK2* (Singleton and Hardy, 2019). Studies of PD genetics have revealed some of the mechanisms including abnormal α-syn deposition, oxidative stress, mitochondrial dysfunction, and abnormalities in the ubiquitin-proteasome system leading eventually to cell death (Ganguly et al., 2021). Genome-wide association studies (GWASs) have identified many single nucleotide polymorphisms (SNPs) as risk factors for PD (Nalls et al., 2014; Blauwendraat et al., 2020). However, it is still unclear how and to what extent each of these genes is related to PD pathology.

*GBA* has recently been recognized as a risk gene for PD. *GBA* is the causative gene of Gaucher’s disease, an autosomal recessive lysosomal disorder caused by glucocerebrosidase (GBA) deficiency (Hruska et al., 2008). It is reported that decreased GBA activity increases soluble α-syn oligomers (Mazzulli et al., 2011). Interestingly, decreased GBA activity in the brain is also seen in patients with sporadic PD (Gegg et al., 2012). The OR for any *GBA* mutation in PD patients vs. controls was reported to be 5.43 (Sidransky et al., 2009). Carriers of *GBA* mutations are reported to represent 4–29% of patients with PD, and *GBA* may be related to the early onset of PD (Lees et al., 2009).

### Fluid Biomarkers

There are few established serum biomarkers for PD. However, several reports have pointed out the association between PD and inflammatory markers. For example, a study found a correlation between the development of sporadic PD and elevated inflammatory biomarkers such as serum TNF-α and IL-6 (Ferrari and Tarelli, 2011). Another study found that decreased apolipoprotein A1 and increased C-reactive protein (CRP) were associated with severe motor symptoms, depression, and sleep disorders (Ferrari and Tarelli, 2011; Lawton et al., 2020).

The most straightforward fluid biomarker should be the direct measurement of α-syn. However, there are some difficulties in measuring the protein in body fluids. Blood has the advantage of easy collection, but simple measurement of α-syn using a blood sample is not currently helpful in distinguishing PD from healthy population. Although there are multiple reports of elevated plasma or serum α-syn levels in PD patients compared with healthy people (Chang et al., 2020), reports on the correlation between α-syn levels and clinical symptoms are inconsistent. There is even a report of decreased plasma α-syn in PD patients (Li et al., 2007). Technical problems may underlie the discrepancies, including contamination of α-syn from erythrocytes due to hemolysis, differences in assay technique, and differences in the binding ability of the antibodies to α-syn (Ganguly et al., 2021). More recently, α-syn oligomers and aggregated α-syn have drawn attention as biomarkers for the progression of PD (Maass et al., 2019). The α-syn oligomers and aggregated α-syn may be involved in the transcellular transmission of α-syn (Okuzumi et al., 2021).

Measurement of α-syn in CSF should be established. Many studies have reported that total α-syn is lower in patients with PD than in healthy controls (Tokuda et al., 2006; Mollenhauer et al., 2013; Wennström et al., 2013). However, the specificity of this finding is not high enough to distinguish PD from healthy controls or from other parkinsonian syndromes (Zhou et al., 2015). Measurement of phosphorylated α-syn or the ratio of phosphorylated α-syn to total α-syn might be helpful in distinguishing PD from healthy controls and from other parkinsonian syndromes (Majbour et al., 2016). CSF studies also show an increase in α-syn oligomers and an increase in the ratio of α-syn oligomer to total α-syn in PD (Tokuda et al., 2010; Majbour et al., 2016; Kakuda et al., 2019). The development of measurement methods has seen recent progress (Kakuda et al., 2019; Okuzumi et al., 2021). Recently, real-time quaking-induced conversion (RT-QuIC), a technique originally developed to detect the self-aggregating ability of proteins in prion diseases, was used for detecting α-syn aggregation with a sensitivity of 92% (DLB) and 95% (PD), and a specificity of 100% (Fairfoul et al., 2016). The use of the novel assays, including RT-QuIC and α-syn oligomers, currently promises the most useful biomarker to precisely capture synuclein deposition.

### Neuroimaging

#### Blood Flow/Metabolism Changes

Changes in cerebral perfusion are used primarily for the auxiliary diagnosis of PDD. Using SPECT, decreased perfusion is observed along with disease progression and impaired cognitive function in patients with PDD. In particular, perfusion in the frontal, parietal and occipital lobes is reduced in patients with PDD (Spampinato et al., 1991; Sawada et al., 1992). Furthermore, in PDD patients with impaired visual cognitive function, decreased perfusion in the occipital lobe is remarkable (Abe et al., 2003). In FDG-PET, glucose hypermetabolism is observed mainly in the putamen, motor cortex and cerebellum, and hypometabolism is seen in the posterior temporoparietal and occipital lobes (Meyer et al., 2017; Thobois et al., 2019). An FDG-PET study revealed that DLB had higher glucose metabolism in PCC and precuneus than did AD, irrespective of amyloid deposition (Graff-Radford et al., 2014).

#### Positron Emission Tomography/Single-Photon Emission Computed Tomography to Detect Monoaminergic Dysfunction in Parkinson’s Disease Spectrum

Single-photon emission computed tomography can visualize PD-derived neurodegeneration, such as the reduction in dopaminergic nerve endings and the loss of the epicardial sympathetic nerve.

^123^I-meta-iodobenzylguanidine (MIBG)-SPECT captures the loss of postganglionic sympathetic neurons in the PD spectrum. It was reported that abnormalities in ^123^I-MIBG-SPCET could be diagnostic in PD, with a sensitivity of 84.3% and a specificity of 89.5% (Sawada et al., 2009). ^123^I-MIBG-SPECT abnormality can be observed at the early stages, even before the onset of definitive motor symptoms in PD (Heinzel et al., 2019). However, it should be noted that some patients can show a false negative finding in the early stages (Sawada et al., 2009).

Presynaptic dopaminergic neuronal loss can be detected using dopamine transporter (DAT)-SPECT. Of the many radiotracers, ^123^I-Ioflupane is the easiest to access. There are multiple reports that the sensitivity and specificity for loss of dopaminergic nerve terminals using DAT-SPECT are 90% or higher (Pirker, 2003; Brigo et al., 2014; O’Brien et al., 2014). Still, false-negative cases have been pointed out (Ba and Martin, 2015). Although it is challenging to distinguish PD from other parkinsonian syndromes using the results from DAT-SPECT only, some studies reported that visual inspection and evaluation of specific binding ratio (SBR) over time can help distinguish PD from other synucleinopathies (Sakakibara et al., 2020). It has been reported that the combined use of DAT-SPECT and MIBG SPECT enhances the specificity of the diagnosis of PD (Yoshii et al., 2017). Therefore, if clinical diagnosis of PD is challenging, the combined use of DAT-SPECT and MIBG SPECT may be considered. Semi-quantified values are available in the form of heart-mediastinal ratio in MIBG-SPECT and SBR of DAT-SPECT. Furthermore, a correction method of these values across different facilities has been proposed for multicenter research (Nakajima et al., 2012; Matsuda et al., 2018).

#### Positron Emission Tomography to Detect Abnormal Protein Deposition in Parkinson’s Disease Spectrum

The PET imaging technology for visualizing α-syn accumulation has not been established yet (Verdurand et al., 2018). Several PET tracers are under development, and animal experiments have confirmed the affinity of the tracers for α-syn aggregates (Kuebler et al., 2021). If an α-syn tracer is developed for humans, it will be possible to identify patients in the preclinical/prodromal stage, to evaluate changes in α-syn deposition in the course of disease progression, and to distinguish PD from other parkinsonian syndromes. Further research and development are urgently needed.

In terms of Aβ/tau accumulation, patients with PDD/DLB have many similarities with AD from multiple perspectives. In a cross-sectional amyloid PET study, PDD and DLB showed moderate to prominent amyloid burden in most cases whereas PD with normal cognition had little amyloid burden (Mashima et al., 2017). The spatial distribution of amyloid in PDD/DLB was similar to that in AD (Shimada et al., 2013). It was also reported that the uptake pattern of tau PET tracer in the temporal-lobe is different between AD and DLB (Kantarci et al., 2017). This discrepancy between PDD/DLB and AD is still under investigation and is going to be discussed in detail in the UNRESOLVED QUESTIONS AND FUTURE DIRECTIONS section.

#### Structural Magnetic Resonance Imaging

Previously in the PD research, structural MRI was performed only to rule out other parkinsonian syndromes such as vascular parkinsonism. This poor performance was partly due to the difficulty in detecting PD-specific structural abnormalities using conventional sequences with low-field MRI imaging below 1.5 T (Sterling et al., 2016). With a higher magnetic field of 3 T, group-level analyses revealed a loss of volume in the putamen (Tinaz et al., 2011), midbrain, basal ganglia, basal forebrain and medial temporal lobe (Zeighami et al., 2015), occipital lobe and head of the caudate nucleus (Sterling et al., 2013; Lewis et al., 2016). As symptom–structure correlates, motor symptoms have been associated with frontal lobe atrophy (Burton et al., 2004; Rosenberg-Katz et al., 2013), and occipital lobe atrophy with hallucinations (Pagonabarraga et al., 2014). Cognitive impairment is related to atrophy of widely distributed structures (Zeighami et al., 2019), including the hippocampus (Laakso et al., 1996; Camicioli et al., 2003; Junqué et al., 2005; Tam et al., 2005; Ibarretxe-Bilbao et al., 2008), the anterior cingulate cortex (Nagano-Saito et al., 2005), the temporal cortex (Summerfield et al., 2005), the frontal cortex (Burton et al., 2004; Nagano-Saito et al., 2005), and the parietal cortex (Sanchez-Castaneda et al., 2009).

The substantia nigra pars compacta (SNc), where the cell bodies of the nigrostriatal dopamine neurons reside, is critical in the clinical evaluation of PD. Recently, technology with high-field MRI of 3 T or higher has allowed visualization of pathological processes in the SNc (Wang et al., 2016). For the assessment of the SNc, susceptibility-weighted imaging (SWI), quantitative susceptibility mapping (QSM) and neuromelanin (NM) imaging may have a diagnostic value. SWI is sensitive to the iron content in the brain. In patients with PD, iron is accumulated in the brain; therefore, iron-deposition imaging may be useful. SWI studies suggest that iron deposition is higher in patients with PD than in healthy people in the SNc, caudate nucleus, red nucleus, putamen and globus pallidus (Zhang et al., 2009; Wu et al., 2014; Wang et al., 2016). QSM is another iron mapping technique through the modeling of magnetic susceptibility sources. QSM studies indicate increased iron deposition in the SNc (Ahmadi et al., 2020; Poston et al., 2020), red nucleus (Cheng et al., 2020), prefrontal cortex, caudate (Chen et al., 2019) and putamen (Thomas et al., 2020) in PD. In a meta-analysis of the iron deposition imaging in PD, iron deposition in the SNc was shown with both SWI and QSM. Moreover, positive correlation between QSM iron deposition and the total UPDRS score has been reported (Pyatigorskaya et al., 2020).

Neuromelanin-contrast MRI might provide a promising biomarker for the PD spectrum. NM is present in dopaminergic neurons in the SNc. Turbo spin echo T1-weighted MRI sequence with magnetization transfer (Sasaki et al., 2008) and gradient recalled echo with magnetization transfer (van der Pluijm et al., 2021) are currently used to yield a contrast sensitive to NM. NM MRI signals increase with age in the healthy population, but are markedly decreased in PD, reflecting the loss of dopamine neurons (Sasaki et al., 2008; Zucca et al., 2014).

#### Diffusion Tensor Imaging

In PD, many studies have reported correlations between decreased FA in the SN and severe motor symptoms (Prakash et al., 2012; Schuff et al., 2015; Langley et al., 2016). FA abnormalities have been reported also for non-motor symptoms, and these are associated with cognitive symptoms, mood disorders, hyposmia, hallucinations, and REM sleep behavior disorder (RBD) (Hall et al., 2016).

Patients with PDD show decreased FA and increased mean diffusivity throughout the cerebral white matter compared with healthy people (Melzer et al., 2013). Reduced hippocampal FA is also reported in patients with PDD compared with patients with PD and those with normal cognitive functions (Chen B. et al., 2015). Others have reported an association of general cognitive decline evaluated by MMSE and MoCA with lower FA in the corpus callosum, the anterior cingulate cortex, and the frontal white matter regions (Kamagata et al., 2012; Chen B. et al., 2015).

#### Functional Magnetic Resonance Imaging

In PD, various alterations are reported in motor-related resting-state networks, including the basal ganglia (Helmich et al., 2010; Hacker et al., 2012; Agosta et al., 2014; Rolinski et al., 2015), cerebellum (Hu et al., 2015; O’Callaghan et al., 2016) and SMN (Fling et al., 2014; Bell et al., 2015; Canu et al., 2015) (Figure 1B). A meta-analysis suggests that decreased FC in the posterior putamen in PD may be a consistent finding (Herz et al., 2014).

Changes in FC may reflect a specific type of motor symptom. Tremor-dominant PD patients, compared with patients without tremor, show increased FC within the striatum (Dirkx et al., 2016), between the subthalamic nucleus and motor and primary somatosensory cortices (Baudrexel et al., 2011), and among the cerebellum, thalamus and motor cortex (Helmich et al., 2011). Nevertheless, decreased FC in tremor-dominant PD has also been reported in the cerebellum (Hu et al., 2015), putamen (Chen H.M. et al., 2015) and primary somatosensory cortex (Zhang et al., 2015). Akinetic-rigid subtype patients, compared with healthy controls, show increased FC in the left putamen, bilateral angular gyri, bilateral medial prefrontal cortices (MPFC) (Zhang et al., 2015) and anterior DMN (Hou et al., 2018b), while they show decreased FC in the precuneus, amygdala (Guan et al., 2017), the left inferior parietal cortex and PCC within the DMN (Karunanayaka et al., 2016; Hou et al., 2018b), and the precentral gyrus (Hu et al., 2015). In summary, the akinetic-rigid subtype shows more widespread alterations in FC, especially in the DMN, compared with the tremor-dominant subtype. This finding may be related to the observation that patients with the akinetic-rigid subtype are more likely to develop cognitive impairment than those with the tremor-dominant subtype (Karunanayaka et al., 2016). Freezing of gait (FOG) is a PD-related gait disturbance characterized by sudden and temporary inability to walk forward or turn (Vandenbossche et al., 2012). A study reported a correlation between severer FOG and lower FC in the SMN (Canu et al., 2015) whereas another study showed increased FC in the SMN in patients with PD-FOG (Fling et al., 2014).

Functional connectivity alterations in PD vary depending on the disease stage. Patients with early drug-naive PD show altered functional connectivity in the cerebello-thalamo-cortical network (Hou et al., 2018a). Similar changes were reported in several prodromal PD studies before the onset of motor symptoms (Rolinski et al., 2016; Yamada et al., 2019; Wakasugi et al., 2021). Focusing on the subsequent changes over time in disease progression, it has been reported that the temporal progression of PD motor symptoms is correlated with reduced FC between the anterior putamen and the midbrain and increased FC between the cerebellum and the motor cortex (Manza et al., 2016). Furthermore, a study suggested that advanced PD patients may show decreased FC between the putamen, caudate and midbrain (Hacker et al., 2012). In terms of treatment, it is known that levodopa, the most commonly used medication for PD, increases motor network connectivity, which is opposite to the changes associated with the progression of motor symptoms in PD (Wu et al., 2009; Esposito et al., 2013; Agosta et al., 2014). A relationship between FC and cognitive symptoms has also been reported.

Patients with PDD show impaired FC of the DMN, compared with PD patients without dementia and healthy participants (Gorges et al., 2015). Decreased DMN-FC in PDD was also reported in a meta-analysis (Wolters et al., 2019). Patients with PDD also showed decreased ECN-FC (Filippi et al., 2019), and similar abnormality was reported at the prodromal stage (Wakasugi et al., 2021) (Figure 1B). Further reports showed disrupted connectivity in the frontoparietal network (Amboni et al., 2015; Borroni et al., 2015) and dorsal attention network (Baggio et al., 2015) in PDD. While a correlation between FC reduction in motor networks and reduced α-syn in the CSF has been reported (Campbell et al., 2015), this finding should be interpreted with caution because reliable CSF biomarkers for PD are not yet established.

In summary, rsfcMRI studies in PD have yielded some consistent findings, such as decreased FC in the putamen in most PD cases and DMN in cognitively impaired PD cases. Further research is needed to clarify the connectivity changes in many other networks, particularly as the observed connectivity changes in a particular network differ among reports. This inconsistency in FC findings may be caused by differences in the predominant clinical symptoms and disease stage (Hohenfeld et al., 2018; Filippi et al., 2019; Wolters et al., 2019). In this regard, it remains unclear if increases in FC reflect a compensatory mechanism or pathological processes. The inconsistency among previous rsfMRI studies may also be ascribed to technical issues: insufficient pre-processing, modulation of FC by medications including levodopa (Tahmasian et al., 2015), and insufficient sample size (Chen et al., 2018). These problems should be overcome by accumulating high-quality rsfMRI data with sufficiently large sample size, followed by cutting-edge preprocessing such as Human Connectome Project (HCP)-style MRI protocol (Glasser et al., 2016) and the adjustment of medication.

### Summary

Till now, AD and PD have been studied independently, and most findings have been obtained independently.

In AD, memory impairment identified by clinical interview and neuropsychological testing is the core clinical symptom. Responsible genes for familial cases and risk genes for sporadic cases have been found, but more are yet to be identified. The identification of reliable biological or imaging markers for sporadic cases is of great importance in AD research to advance the development of disease-modifying therapies. Blood biomarkers are under development, and promising methods have been reported (Nakamura et al., 2018). Measurements of CSF can reveal alterations in key proteins (Aβ and tau) underlying AD pathology. To visualize the spatial distribution of Aβ, amyloid-PET has been established for its utility in clinical studies. A few tracers have been proposed for tau-PET. Structural MRI and perfusion SPECT/FDG-PET are used to detect brain atrophy and reduced blood flow/energy requirement, respectively, both of which indicate neuronal loss. The analysis of brain connectivity by rsfcMRI consistently shows abnormality in the DMN. Moreover, ML combined with FC has permitted discrimination between AD and healthy controls with a degree of accuracy.

Parkinson’s disease is characterized by movement disorder possibly complicating cognitive impairment. In cognitive disturbance in PD, executive function is particularly impaired. Many responsible genes for familial cases and risk genes for sporadic cases have been identified. MIBG-SPECT and DAT-SPECT are widely performed to detect the neurodegeneration of monoaminergic neuronal systems, which is a hallmark of PD pathology. Structural MRI with high magnetic field has utility in PD research for revealing the iron deposition and neurodegeneration in the SNc, which are the core pathological changes in PD. Detection methods for the other pathological hallmark, α-syn, are not established yet. Imaging measures of brain atrophy and blood flow/energy requirement show variable findings, depending in part on the motor subtype and presence of cognitive decline. Decreased perfusion is shown by SPECT in PD with cognitive decline. A group analysis using structural MRI revealed cerebral atrophy, especially in patients with substantial non-motor symptoms. Many rsfcMRI studies are reported, but again, the results vary, depending on the subtype and stage. Yet, network abnormalities of the basal ganglia, frontal lobe, and cerebellum, corresponding to movement disorders, and DMN/frontal lobe abnormalities corresponding to cognitive disorders have been consistently reported.

Because AD and PD are regarded distinct and independent disorders with different core clinical symptoms (cognitive vs. motor), different neuropsychological tests and imaging methods have been traditionally applied to each disease. Unfortunately, different evaluation methods have been applied to symptoms common to AD/PD as well, including cognitive and psychiatric symptoms. Therefore, it remains unclear which pathophysiological mechanisms are common, and which are unique to each disorder, despite the shared symptoms and the putatively common pathological mechanisms.

## Unresolved Questions and Future Directions

Research on AD and PD has substantially progressed in the last few decades, but major questions remain unresolved. Given the overlaps between AD and PD in many aspects, the commonalities and differences between AD and PD need to be clarified, especially from the viewpoints of clinical manifestation, proteinopathy, vasculopathy, and neurocircuitopathy. To this end, it is necessary to construct a cohort containing both AD spectrum and PD spectrum. Related approach has been recently proposed for nation-wide cohort studies including AD and other dementia in Canada and United Kingdom (Chertkow et al., 2019; Morton et al., 2019; Koychev et al., 2020).

### Clinical Viewpoints

Now, it is recognized that patients in the PD spectrum may show AD-like symptoms, and those in the AD spectrum could also exhibit PD-like symptoms, including anosmia and movement disorders. Anosmia is present in both AD and PD. Patients with AD were reported to perform worse than patients with PD in an odor recognition task (Lehrner et al., 1997). Anosmia in AD is common in cases with Lewy body accumulation in the postmortem brain (Olichney et al., 2005). However, it is not clear how α-syn, which constitutes Lewy bodies, is involved in olfaction problems in AD, in contrast to PD, in which neuronal loss occurs in the locus coeruleus, the raphe nuclei, and the nucleus basalis of Meynart (Doty, 2012).

In contrast to the common clinical conception, patients with PD may present with AD-like memory-dominant cognitive decline (Das et al., 2019). Conversely, some patients with AD may display parkinsonism (Sasaki, 2018). In a longitudinal study of AD with a mean follow-up of 3.6 years, 12.3% of patients had clinically apparent parkinsonism at the initial visit, and 22.6% at the final visit (Portet et al., 2009). Interestingly, patients with AD complicated with mild parkinsonism exhibit decreased DAT in the caudate nucleus, the pattern of which is closer to DLB than PD (Chung et al., 2019). To our knowledge, no study has directly examined prodromal PD pathology in AD with motor symptoms using CSF-α-syn or α-syn-PET. BPSD with hallucinations, suggestive of monoamine system dysfunction, a hallmark of PD pathology, is also present in AD.

Similarities in clinical manifestations are clearly seen between AD and PD. Anosmia may point to the common pathophysiology underlying the two. However, since few, if any, large-scale studies used a common clinical scale to compare AD and PD, it is unclear exactly how similar or different are these common symptoms in the two disorders.

### Proteinopathy Viewpoints

Mounting evidence indicates α-syn pathology in the AD spectrum and Aβ/tau pathology in the PD spectrum. In animal experiments, many forms of interactions between AD-related proteins and PD-related proteins are reported, and such molecular interactions may explain the mixed AD and PD pathology common in humans. It has been suggested that Aβ directly affects the toxicity of α-syn. Aβ-rich AD-transgenic mice, including PSEN1/2 and APP mutation, show rapid and widespread aggregation of α-syn (Bassil et al., 2020). This finding suggests that Aβ plaques may promote seeding and spreading of α-syn. Tau and α-syn directly interact with each other to promote co-assembly (Bassil et al., 2021). Thus, each abnormal protein promotes the formation of other abnormal proteins.

Most postmortem brains in patients with PDD are complicated by varying degrees of AD pathology (Dickson et al., 2017). Thus, it is widely accepted that a certain degree of PD cognitive impairment may be associated with AD pathology. In AD, however, approximately 33% of the patients exhibit significant Lewy body pathology alongside Aβ (Deture and Dickson, 2019), especially the presence of Lewy pathology in the olfactory bulb and the amygdala in patients with AD (Serrano-Pozo et al., 2011; Deture and Dickson, 2019). Also, patients in the PD spectrum show accompanying AD pathology (Kosaka and Manabe, 2010). A multicenter-cohort study of DLB examined Aβ and p-tau with CSF and PET. Among the patients, 32% were Aβ positive, 13% were p-tau positive, and 15% were positive for both Aβ and p-tau (Ferreira et al., 2020). Aβ and p-tau were found to be independent risk factors for cognitive decline in DLB. Interestingly, in the same study, p-tau was associated with a lower likelihood of parkinsonism and RBD in DLB.

While amyloid pathology in PDD/DLB is common, the complications of amyloid pathology in non-demented PD remain unclear. In a study using amyloid-PET, the prevalence rate of Aβ was rather low in non-demented PD patients compared with the healthy population (Mashima et al., 2017). It is possible that the involvement of Aβ pathology may be different in non-demented PD and PDD/DLB, but further studies are needed.

Because interactions of abnormal proteins in advanced stages of AD/PD-related dementia may occur, it is important to examine overlapping or interacting proteinopathies even in the preclinical and prodromal stages. In aMCI, which is considered a prodromal stage of AD, patients show cognitive decline that correlates with increased CSF α-syn levels (Twohig and Nielsen, 2019). Although few studies have evaluated protein interactions in prodromal PD, the coexistence of Aβ and tau pathology may promote the progression of prodromal PD to DLB (Berg et al., 2021). Protein interactions in prodromal AD and PD remain unclear, and this requires research attention. Considering that the therapeutic target of each disease is shifting to the early stage, a more thorough understanding of the disease, including protein interactions in the preclinical/prodromal stage, is urgently needed.

### Vasculopathy Viewpoints

Cerebrovascular lesions cause cognitive or motor impairment. However, the significance of vascular lesions including white matter hyperintensities (WMHs) is often underestimated in the pathogenesis of dementia in comparison with, e.g., the proteinopathy viewpoint (Prins and Scheltens, 2015). In the AD spectrum, multiple studies indicated the presence of vascular lesions as an important background pathology (Prins and Scheltens, 2015; Saito et al., 2015; Muddapu et al., 2020). Decreased amyloid clearance has been proposed for the involvement of WMHs in AD pathogenesis (Saito et al., 2015). WMHs are also considered to underlie dementia in PD (Bohnen and Albin, 2011; Liu et al., 2021).

### Neurocircuitopathy Viewpoints

In AD, many abnormalities in connectivity centering on the hippocampus and DMN, related to Aβ pathology, have been reported (Takamura and Hanakawa, 2017). In PD, abnormalities in the motor networks, including the basal ganglia and SMN, have been consistently reported. Importantly, DMN abnormalities have been observed in both the AD and PD spectra with cognitive impairment. AD features abnormalities mainly in the precuneus and PCC within the DMN (Ibrahim et al., 2021) whereas DLB has more widespread network abnormalities involving the DMN and the occipital lobe (Wolters et al., 2019).

It seems important to investigate the relationship between proteinopathic abnormalities and neuropsychological indices in the AD and PD spectra, and their relationship with the neurocircuitopahty viewpoint, including the DMN abnormality. Existing MRI cohort studies do not allow us to perform such an analysis because they recruit AD and PD cohorts independently with different evaluation metrics. Therefore, it remains unknown how Aβ, tau and α-syn are involved in the differences in DMN abnormalities between AD and PD. By performing network analysis using biomarkers of both AD and PD, it may be possible to assess the degree to which each AD/PD pathology contributes to network changes. In particular, little is known about the relationship between PD network abnormalities and proteinopathy.

### Summary

Although the presence of substantial overlaps has been demonstrated, AD and PD cohorts have been recruited and evaluated separately. In particular, there is a marked discrepancy between the evaluation metrics for cognitive and neuropsychiatric functions, which limits the utility of existing cohorts for comparing AD and PD. Therefore, we recommend that a new cohort should be designed to resolve this problem. Each AD/PD spectrum should be evaluated throughout the preclinical, prodromal, early, and advanced stages.

Accumulating evidence indicates that the comorbidity of AD and PD pathology is common in elderly people. Many questions remain unanswered. For example, it is not yet known if the comorbidity is simply coincidental to the aging process *per se* or if it is caused by the interactions of the key common molecules in AD and PD. It is not clear when and how the mixed AD and PD pathology (i.e., co-pathology) emerges and progresses. Furthermore, it is unclear how the co-pathology affects clinical symptoms, brain structure, and circuit pathology.

To answer these questions, we should first consider merging data from existing cohorts targeting a single disease spectrum for efficiency. There are renown MRI cohorts including ADNI and PPMI (Petersen et al., 2010; Marek et al., 2011; Weiner et al., 2017; Veitch et al., 2019), which have evaluated the AD and PD spectrum separately in depth. For biomarker/genetic studies, it may be worthwhile evaluating a common set of proteins or risk genes from originally distinct cohort. However, it is known that technical differences such as a way to collect, store and measure samples may limit the reliability of the results. For imaging studies, it is still difficult to perform accurate statistical analysis of MRI data obtained from different MR scanners and protocols (Duchesne et al., 2019). Although image analysis methods to remove the scanner difference have been proposed, such methods will remove the difference across the disease spectra when AD is heavily sampled in some sites and PD is heavily sampled in the others. Also, since each cohort tend to focus on spectral-specific clinical and neuropsychological indices, it is difficult to compare network indices correlated with a common set of neuropsychological indices. Recently, some PD cohorts employed amyloid and tau PET. It is intriguing to compare spatial distribution of pathological protein across spectra; however, it is still difficult to associate the findings with neuropsychiatric indices with different batteries.

**FIGURE 2:**
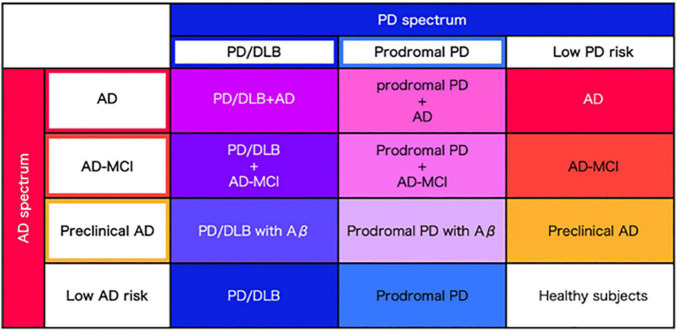
Precise classification of AD and PD spectrum. Regarding AD and PD as spectra, precise disease classification will be performed based on the severity of abnormal protein accumulation and clinical data, derived from each AD and PD spectrum.

Considering the homology of AD and PD spectra in terms of abnormal proteins, vascular factors, motor sand cognitive symptoms, it is most reasonable to perform the same elaborate evaluation in a single cohort employing the same evaluation measures across the spectra. Here we propose to establish a newly designed cohort combining both the AD and PD spectra (Figure 2). Then, symptoms, biomarkers, brain structure and circuit pathology should be evaluated. The unified AD and PD cohort is expected to overcome the limitation of the existing AD cohort and PD cohort combined.

To investigate the similarities and differences between AD and PD, it is important to design a cohort study with a few key characteristics. First, it is necessary to apply the same behavioral, cognitive, and neuropsychiatric scales to AD and PD. Behaviorally, because parkinsonism is sometimes seen in AD, it may be needed to evaluate motor functions of both AD and PD with MDS-UPDRS. CDR and ADAS, which are used for AD, and FAB and MoCA, which are used for early PD, may need to be jointly used. It would be interesting to compare the results between PD-MCI in AD-MCI with tests for the five cognitive domains. Unified questionnaire must be used for assessment of behavioral and psychiatric symptoms such as anxiety, depression, and sleep problems, which are common to both AD and PD. The same olfactory test should be applied to AD and PD to test anosmia.

Next, Aβ/p-tau and α-syn should be evaluated for both AD and PD. To date, Aβ and p-tau have been mostly measured for AD only, and α-syn has been measured for PD cohorts only. The same assay methods of these proteins should be applied to blood and spinal fluid obtained from AD and PD. In postmortem brain research, NFTs appear in the locus coeruleus and entorhinal cortex first, and then spread to the limbic system and neocortex as dementia progresses (Braak and Braak, 1991). To evaluate the spatial distribution of abnormal protein accumulations over time, longitudinal PET imaging is important.

Finally, network and structural abnormalities of the brain should be compared in detail, preferably using the HCP-style MRI protocol (Glasser et al., 2016; Koike et al., 2021). This modern MRI technology should be combined with the standardized assessment of clinical scales and proteinopathies with fluid biomarkers and SPECT/PET. The effects of Aβ, tau and α-syn accumulation on network connectivity and their association with symptoms should be examined.

Here are cases where the unified AD and PD cohort may provide new knowledge. Clinically, aMCI is often presumed to have AD pathology (Petersen et al., 1999, 2001). The unified AD and PD cohort will provide data to suggest how common is aMCI without evidence for AD pathology but, for example, decreased DAT indicative of the PD spectrum (presumably prodromal DLB). Then, markers of the circuit pathology of such non-AD MCI may be compared with those of PD-MCI “neuroimaged” in the cohort to examine if the markers of neurocircuitopathy could serve as early markers differentiating AD-MCI and non-AD MCI. In the unified AD and PD cohort, vascular burden as a common background pathology to AD and PD can be evaluated from multiple perspectives, such as interactions of WMH burden with the proteinopathy, symptoms or circuit pathology. We can then ask how WMH burden similarly or differentially modulates manifestation of the AD spectrum and PD spectrum. The unified AD and PD cohort may also clarify the specific roles of the DMN in cognitive decline in the AD spectrum and PD spectrum since DMN is implicated for both AD and PDD.

## Conclusion

Indispensable knowledge has been garnered from previously established AD cohorts and LBD cohorts. Nevertheless, evaluating abnormalities within each disease category only, without knowing the similarities or differences between the two, cannot provide a clear understanding of their pathophysiologies, from protein interactions and disease-spectrum crossovers. Constructing a cohort with multifaceted evaluations for both AD and LBD is not an easy task. However, longitudinal cohort studies, which meet the requirements proposed above, should provide novel insight into the pathophysiologies of AD and LBD. This knowledge will in turn provide much-needed data for guiding the development of disease-modifying therapies targeting the pathological proteins in AD and LBD.

## Author Contributions

NW drafted the manuscript. NW and TH wrote the manuscript. Both authors contributed to the article and approved the submitted version.

## Conflict of Interest

The authors declare that the research was conducted in the absence of any commercial or financial relationships that could be construed as a potential conflict of interest.

## Publisher’s Note

All claims expressed in this article are solely those of the authors and do not necessarily represent those of their affiliated organizations, or those of the publisher, the editors and the reviewers. Any product that may be evaluated in this article, or claim that may be made by its manufacturer, is not guaranteed or endorsed by the publisher.
